# HIV prevention programme cascades: insights from HIV programme monitoring for female sex workers in Kenya

**DOI:** 10.1002/jia2.25311

**Published:** 2019-07-22

**Authors:** Parinita Bhattacharjee, Helgar K Musyoki, Marissa Becker, Janet Musimbi, Shem Kaosa, Japheth Kioko, Sharmistha Mishra, Shajy K Isac, Stephen Moses, James F Blanchard

**Affiliations:** ^1^ Centre for Global Public Health University of Manitoba Winnipeg Manitoba Canada; ^2^ Partners for Health and Development in Africa Nairobi Kenya; ^3^ National AIDS and STI Control Programme Ministry of Health Nairobi Kenya; ^4^ Department of Medicine St. Michael's Hospital University of Toronto Toronto Ontario Canada; ^5^ Institute of Medical Sciences University of Toronto Toronto Ontario Canada; ^6^ Institute of Health Policy Management and Evaluation Dalla Lana School of Public Health University of Toronto Toronto Ontario Canada; ^7^ Karnataka Health Promotion Trust Bangalore Karnataka India

**Keywords:** key population, HIV prevention, Kenya, cascade, monitoring

## Abstract

**Introduction:**

HIV prevention cascades have emerged as a programme management and monitoring tool that outlines the sequential steps of an HIV prevention programme. We describe the application of an HIV combination prevention programme cascade framework to monitor and improve HIV prevention interventions for female sex workers (FSWs) in Kenya.

**Methods:**

Two data sources were analysed: (1) annual programme outcome surveys conducted using a polling booth survey methodology in 2017 among 4393 FSWs, and (2) routine programme monitoring data collected by (a) 92 implementing partners between July 2017 and June 2018, and (b) Learning Site in Mombasa (2014 to 2015) and Nairobi (2013). We present national, sub‐national and implementing partner level cascades.

**Results:**

At the national level, the population size estimates for FSW were 133,675 while the programme coverage targets were 174,073. Programme targets as denominator, during the period 2017 to 2018, 156,220 (90%) FSWs received peer education and contact, 148,713 (85%) received condoms and 83,053 (48%) received condoms as per their estimated need. At the outcome level, 92% of FSWs used condoms at the last sex with their client but 73% reported consistent condom use. Although 96% of FSWs had ever tested for HIV, 85% had tested in the last three months. Seventy‐nine per cent of the HIV‐positive FSWs were enrolled in HIV care, 73% were currently enrolled on antiretroviral therapy (ART) and 52% had attended an ART clinic in the last month. In the last six months, 48% of the FSWs had experienced police violence but 24% received violence support. National and sub‐national level cascades showed proportions of FSWs lost at each step of programme implementation and variability in programme achievement. Hotspot and sub‐population level cascades, presented as examples, demonstrate development and use of these cascades at the implementation level.

**Conclusions:**

HIV prevention programme cascades, drawing on multiple data sources to provide an understanding of gaps in programme outputs and outcomes, can provide powerful information for monitoring and improving HIV prevention programmes for FSWs at all levels of implementation and decision‐making. Complexity of prevention programmes and the paucity of consistent data can pose a challenge to development of these cascades.

## 1 Introduction

Despite the progress made by prevention programmes globally, the decline in new HIV infections among adults has slowed in the past decade 1. According to UNAIDS, HIV prevention services are not being provided on an adequate scale, with sufficient intensity, nor reaching those most in need 2. Combination prevention for key populations (KPs) has been identified as a key pillar in the UNAIDS HIV Prevention Road Map 2020 3. Approximately 40% of new HIV infections in 2017 occurred among KPs including female sex workers (FSWs), men who have sex with men (MSM), people who inject drugs (PWID) and transgender persons 2. Kenya's HIV epidemic is driven by sexual transmission and is generalized, meaning it affects all sections of the population 4. However, a disproportionate number of new infections occur among KPs 4, estimated at 33% of new infections annually in Kenya 5. HIV prevalence in Kenya among FSWs is 29.3%, MSM 18.2% and PWID 18.7% 6 compared to an estimated national adult HIV prevalence of 4.8% 7.

For HIV prevention efforts to have an impact, increased emphasis on evidence‐driven programme design and implementation, strategic and iterative programme monitoring, and rigorous evaluation of outcomes are required 8. Cascade analysis has been used in measuring success in HIV care and this strategy has been translated to HIV prevention programmes recently with the development of HIV prevention cascades 9. A prevention cascade defines important steps in a comprehensive prevention programme, provides estimates of the proportions of populations engaged or lost at each step and provides a framework for planning further actions to improve programmes 10. Prevention cascades take into account the linkage between various programme elements 11 and offer insights for programme implementers and national decision makers on how best to analyse bottlenecks and develop solutions to improve programme outcomes for a more effective HIV prevention response 12.

However, challenges to the design and implementation of prevention cascades are complexity of prevention programmes and the paucity of consistent data. HIV prevention programmes are not as linear as HIV testing and treatment programmes, making it more difficult to define a prevention cascade as a series of sequential steps 12. There is an array of different prevention needs for different populations and the context in which these populations live and work, and the barriers that they experience 13, adding complexity to the definition of prevention processes as a single cascade. Moreover, the monitoring of HIV prevention programmes requires the incorporation of combination prevention approach, capturing behavioural, biomedical and structural interventions outcomes at scale 14. In addition, the use of aggregate level data may mask heterogeneity in population and thus more detailed and nuanced information on specific sub‐populations that are not being reached is required 15. While the cascade analysis helps identify gaps, unless there are consistent processes to understand and analyse the bottlenecks that are causing these gaps, programmes cannot identify opportunities to address them at all levels of programme design and implementation 16. While current HIV prevention cascades use a demand and supply framework 10, 14, we demonstrate the application of an HIV prevention programme cascade to measure globally recommended HIV programme outcomes 17. This involves an embedded process of analysis which deliberately avoids predetermined assumptions for loss along the cascade and instead uses data to identify gaps and further explores opportunities by conducting continuous iterative refinement of interventions to address the gaps identified 16. This approach builds on our team's previous work in India 18 and this paper demonstrates the application of this approach and learnings in the Kenyan context.

In this paper, we use data from the Kenya KP HIV Prevention Programme, led by the National AIDS and STI Control Programme (NASCOP), Ministry of Health, covering 32 out of the 47 counties (sub‐national geographical and administrative units) in the country. KP behaviours are criminalized in Kenya which increases their vulnerability to stigma, discrimination and violence 19. The Kenya KP programme recommends implementation of combination prevention interventions in accordance with global guidance 17, 20. The FSW programme has been scaled up to reach over 156,220 FSWs in 32 counties 21. Since 2018, the KP programme has been using an HIV prevention programme cascade approach on a quarterly and annual basis for monitoring and management 22. The aim of this paper is to describe the application of an HIV prevention programme cascade framework to monitor and improve HIV prevention interventions with FSWs and the utility of this approach for decision‐making at the national, sub‐national and implementation levels in Kenya.

## 2 Methods

### 2.1 Data sources

We used two data sources to characterize HIV prevention programme cascades:

#### 2.1.1 Annual programme outcome surveys

NASCOP has been conducting annual outcome surveys with KPs using polling booth survey (PBS) methodology 23 which is designed to minimize the reporting bias which can be seen with face‐to‐face interview methods 24, 25. A PBS is a group interview method in which participants’ responses are unlinked and anonymous. As previously described elsewhere 23, a two‐stage, stratified cluster sampling methodology was used to recruit FSWs for the PBS. For the first stage of sampling in each study site, hotspots were selected as the primary sampling units (PSUs). A “hotspot” was defined as any physical location where FSWs meet their clients. At the second stage, respondents were randomly selected from the PSUs. We used data from a PBS conducted in 2017 among 4393 FSW in 13 counties 26.

#### 2.1.2 Routine programme monitoring data

Kenya conducted size estimation of FSWs in 2012 in 32 counties using a geographic mapping approach 27 and estimated approximately 133,675 FSWs (range 76,674 to 208,711) 28. Ninety‐two KP implementing partners in Kenya collected routine monitoring data on a monthly basis using standard NASCOP reporting tools 29. Routine programme monitoring data collected by these implementing partners between July 2017 and June 2018 were used to develop national and county level cascades. We also used data from NASCOP Learning Sites 30 for HIV Prevention for FSWs implemented between 2013 and 2014 in Nairobi and implemented between August 2014 and July 2015 in Mombasa to characterize a hotspot‐based programme cascade and sub‐population level programme cascade desegregated by age at implementation partner level respectively.

### 2.2 Data analysis

We used both data sources described above to determine programme inputs, outputs and outcomes to develop national programme prevention cascades. Size estimation data and programme coverage targets, as established by donors to the implementing partners, for the period were used as programme inputs. The coverage target of FSWs as per Kenya AIDS Strategic Framework 2014/15 to 2018‐18 is 90% of estimated FSW population 4 and programme coverage is defined as the proportion of FSWs that receive a defined set of services that address their risk and vulnerability 20. To arrive at the programme outputs, we aggregated the data for the period to arrive at an annual figure and used it as a numerator against the programme coverage targets (the denominator). Following indicators were used: (i) received peer education and contacts; (ii) received condoms; and (iii) received condoms as per estimated need. Condom need was calculated as 76 condoms per month for FSWs on an average, derived from evidence on client volume per month per FSW in 2017. 2017 PBS data were analysed for programme outcomes: (1) behavioural outcomes: (a) condom use at last sex with any paying client and (b) any occasion when had sex with any paying client without using a condom in the last one month; (2) biomedical outcomes: (a) ever tested for HIV; (b) tested for HIV in the past three months; (c) ever enrolled in care (among all FSWs living with HIV (PLHIV)); (d) currently on antiretroviral therapy (ART) (all PLHIV); and (e) ever missed an appointment in the last one month (this indicator was used as a proxy indicator to measure adherence to ART); (3) structural outcomes: (a) arrested or beaten up by police in the last six months and (b) received any support when experienced violence. Similar indicators were used to analyse and develop HIV prevention programme cascades for implementation partners at the hotspot/peer educator level and sub‐population level. Tables S1,S2,S3 show the indicators and data used for analysis.

### 2.3 Ethical approval

Ethical approval was received from the Kenyatta National Hospital‐University of Nairobi Ethical Review Committee, approval number P647/11/2017, to conduct secondary data analysis of the programme monitoring data, including the annual programme outcome surveys (PBS).

## 3 Results

### 3.1 HIV prevention programme cascade at the national level

**Figure 1:**
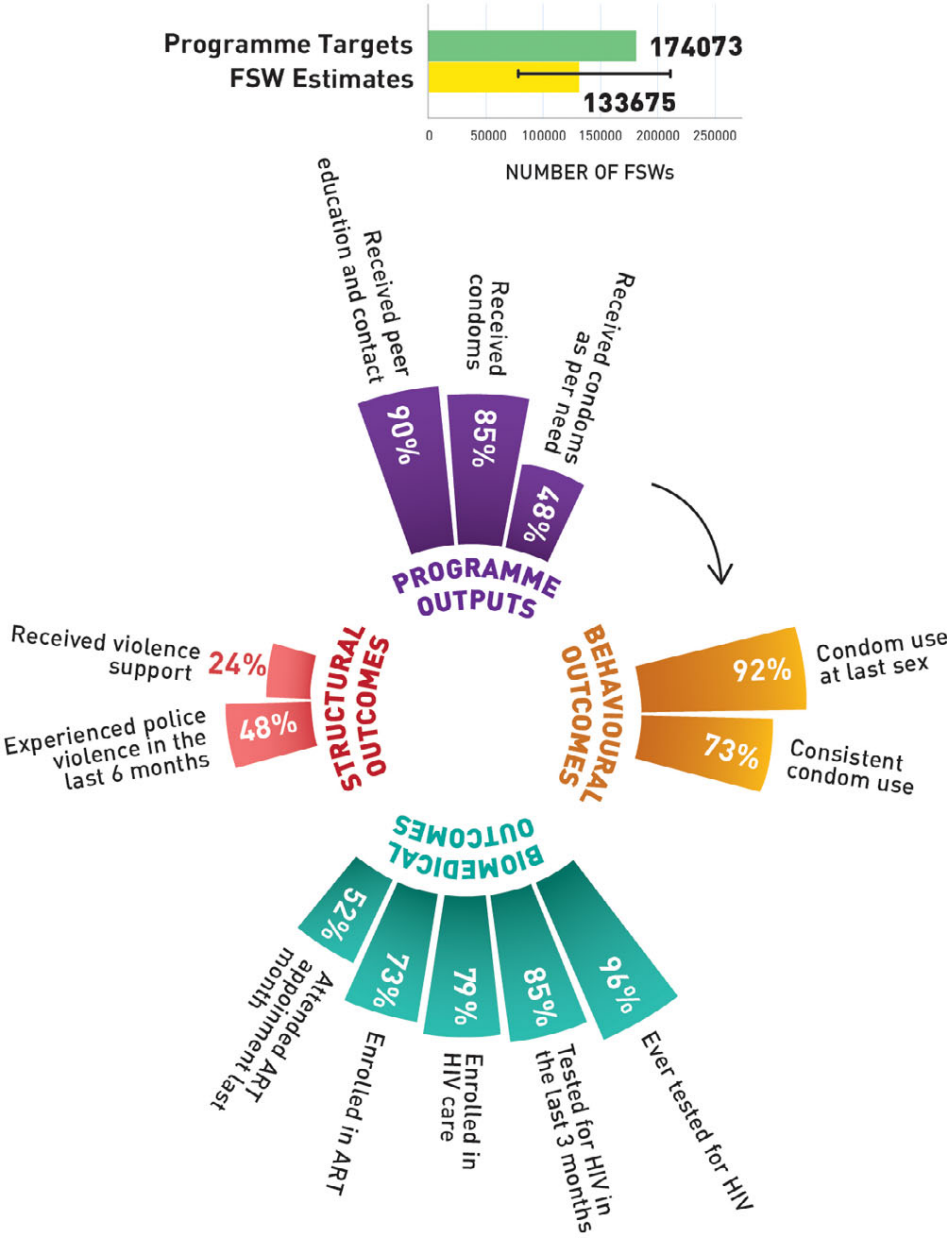
HIV prevention programme cascades, FSW, Kenya FSW, female sex worker; ART, antiretroviral treatment.

Figure 1 shows that the national size estimate of FSWs was 133,675 (range 76,674 to 208,711) and the programme coverage target was 174,073 FSWs. Out of the total programme coverage target, in the year 2017 to 2018, 90% of the FSWs received peer education and contact, 85% of the FSWs received condoms and 48% of the FSWs received condoms as per their estimated need. Although 92% of the FSWs reported using condoms with paying clients in the last sex act, 73% used condoms consistently in the last month. Among the FSW respondents, 96% reported receiving an HIV test in their lifetime, and 85% had tested for HIV in the last three months. Among those FSWs who were HIV positive, 79% were enrolled in HIV care, 73% were currently enrolled with an ART centre and 52% visited the ART clinic in the last one month. While 48% of FSWs had experienced police violence in the past six months, 24% received support to address this violence.

### 3.2 HIV prevention programme cascade at the sub‐national level

**Figure 2:**
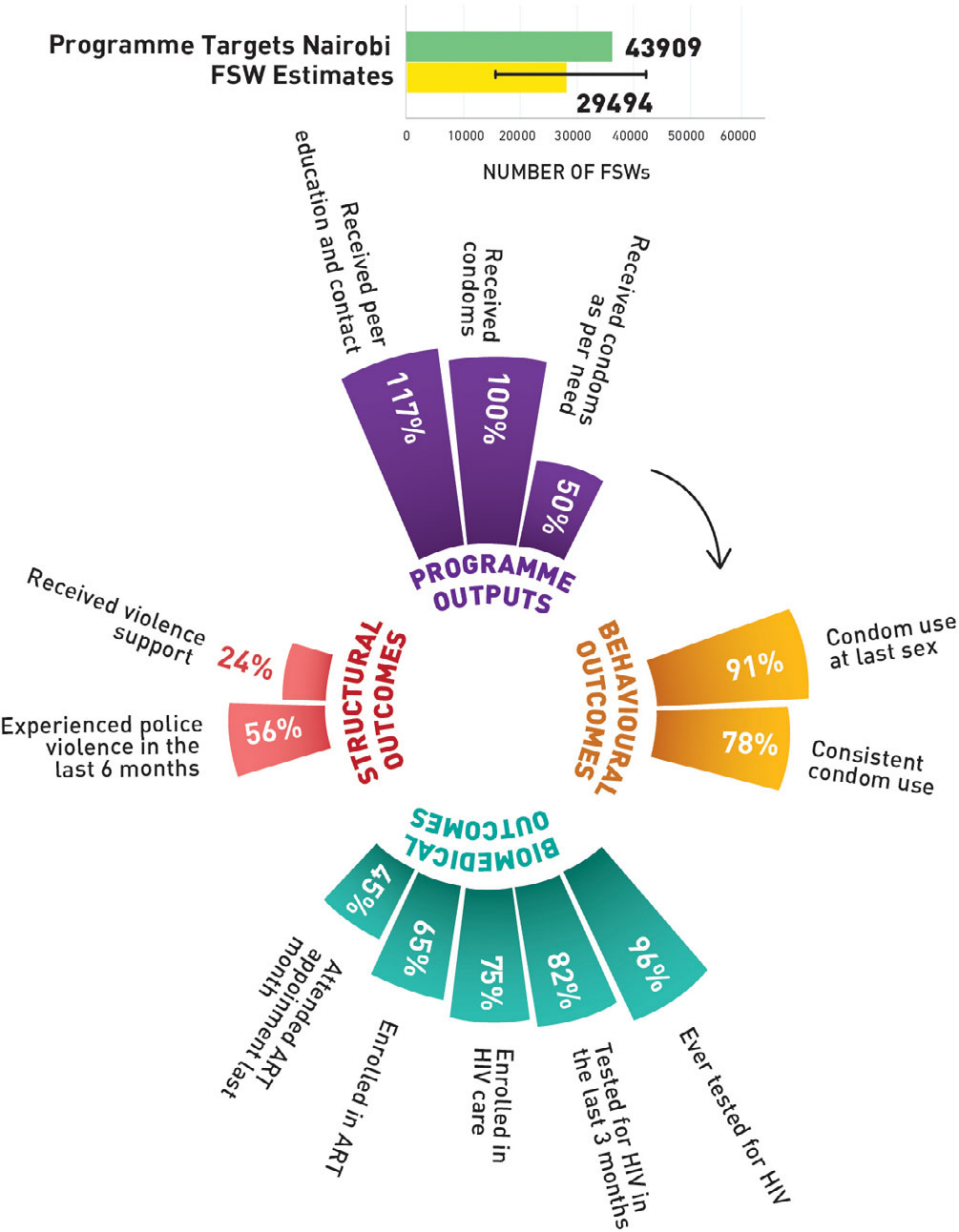
HIV prevention programme cascade for FSW, Nairobi County, Kenya FSW, female sex worker; ART, antiretroviral treatment.

Figures 2 (Nairobi), 3 (Mombasa), 4 (Kiambu) and 5 (Kisumu) present HIV prevention programme cascades across four counties to depict sub‐national variability in cascades. Out of the total programme coverage target in the year 2017 to 2018, 117% of the FSWs in Nairobi, 84% in Mombasa, 74% in Kiambu and 68% in Kisumu received peer education and contact every quarter. All the FSWs in Nairobi, 83% in Mombasa, 72% in Kiambu and 67% in Kisumu received condoms every quarter, and 50% of FSWs in Nairobi, 58% in Mombasa, 33% in Kiambu and Kisumu, respectively, received condoms as per their estimated need. In Nairobi, 91% of the FSWs, 92% in Mombasa, 91% in Kiambu and 92% in Kisumu reported using condoms with paying clients in the last sex act; 78% in Nairobi, 69% in Mombasa, 76% in Kiambu and 84% in Kisumu used condoms consistently in the last month. Among the respondents, 82% in Nairobi, 85% in Mombasa, 89% in Kiambu and 80% in Kisumu had tested for HIV in the last three months and among those who were HIV positive, 75% in Nairobi, 53% in Mombasa, 72% in Kiambu and 90% in Kisumu were enrolled in HIV care, 65% in Nairobi, 47% in Mombasa, 65% in Kiambu and 85% were currently enrolled with an ART centre, and 45% in Nairobi, 27% in Mombasa, 42% in Kiambu and 64% in Kisumu visited the ART clinic in the last one month. Among the FSWs, 56% in Nairobi, 43% in Mombasa, 53% in Kiambu and 31% in Kisumu had experienced police violence in the past six months, and 24% of FSWs in Nairobi, 17% in Mombasa, 27% in Kiambu and 22% in Kisumu received support to address this violence.

**Figure 3:**
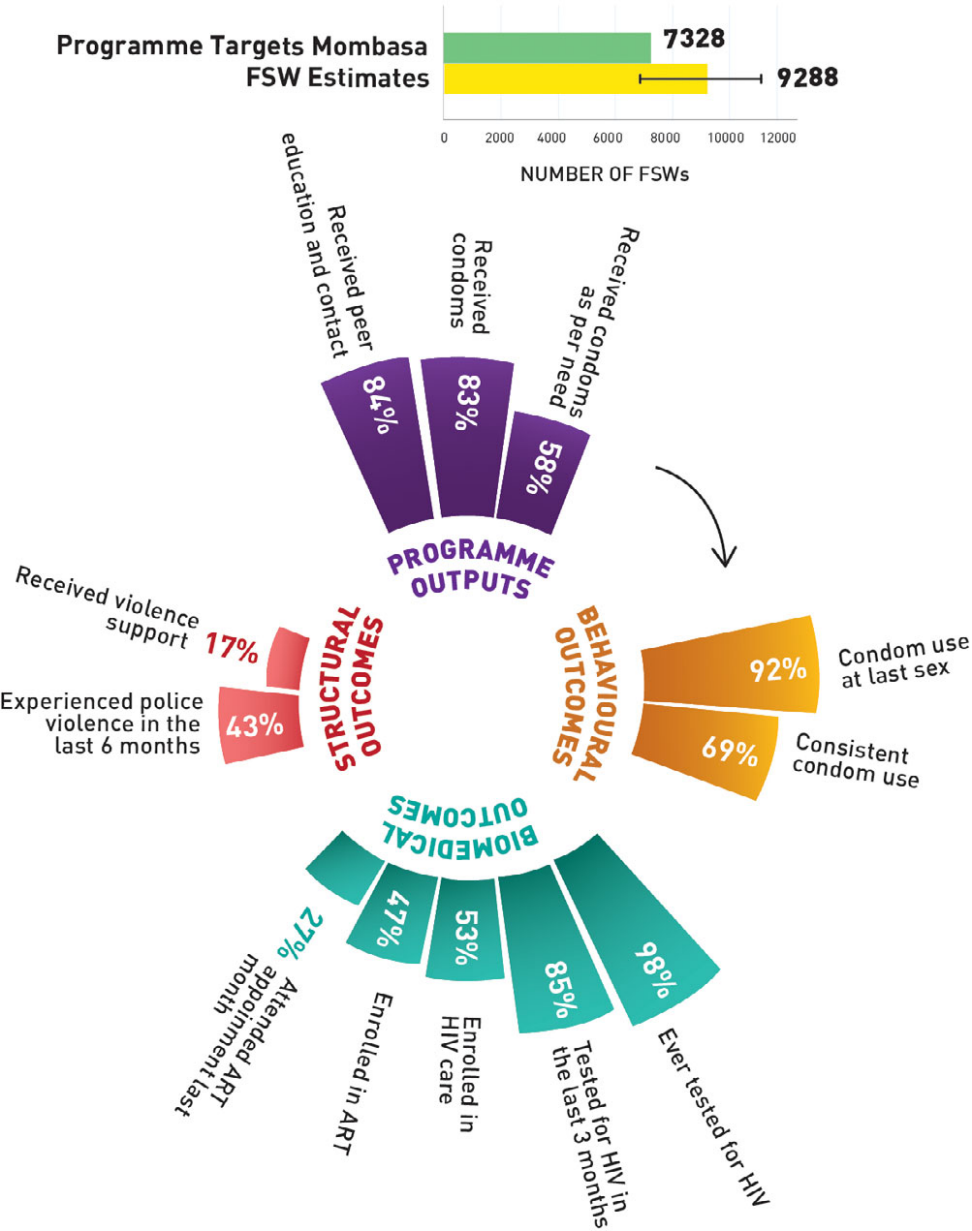
HIV prevention programme cascade for FSW, Mombasa County, Kenya FSW, female sex worker; ART, antiretroviral treatment.

**Figure 4:**
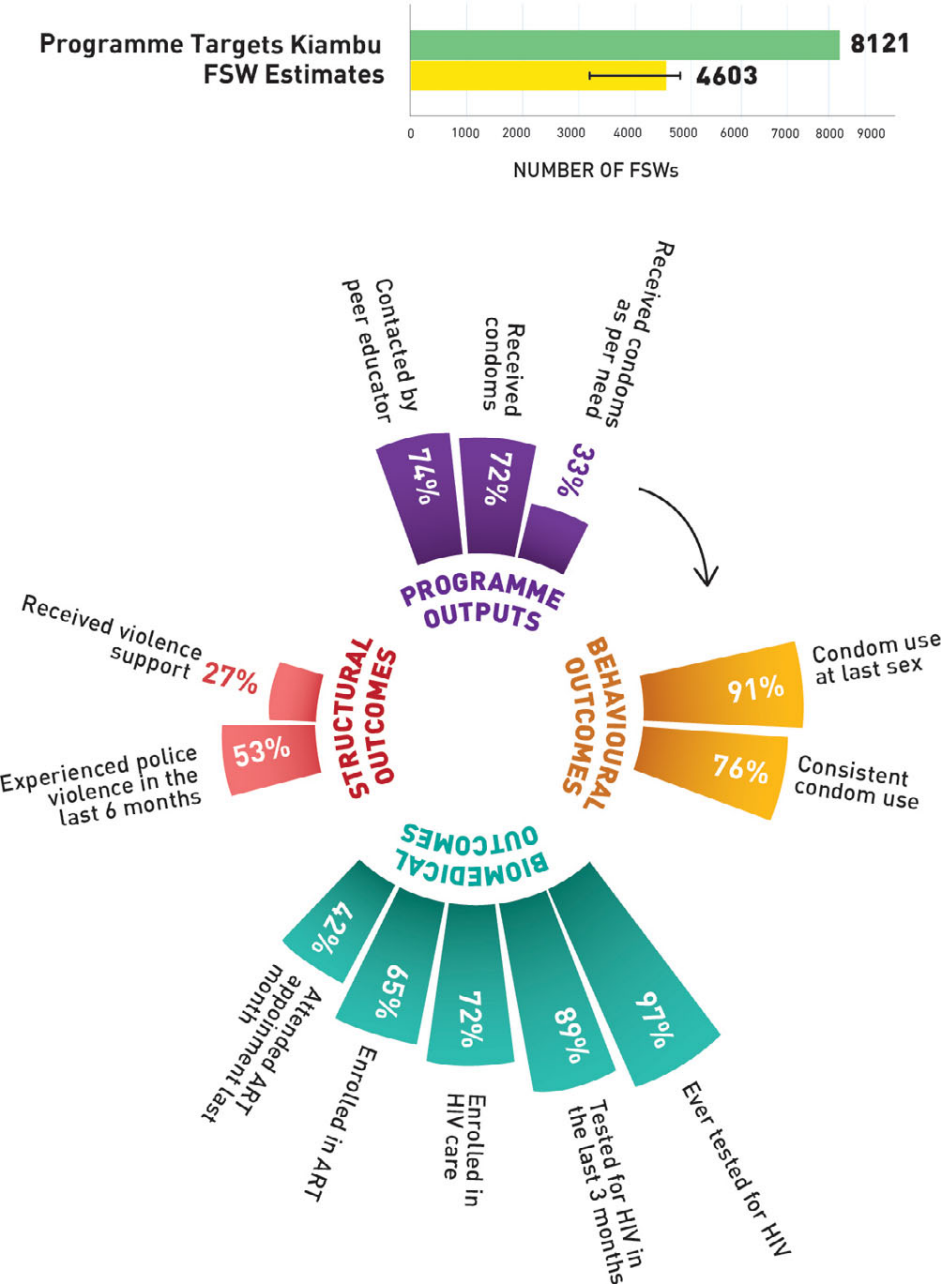
HIV prevention programme cascade for FSW, Kiambu County, Kenya FSW, female sex worker; ART, antiretroviral treatment.

**Figure 5:**
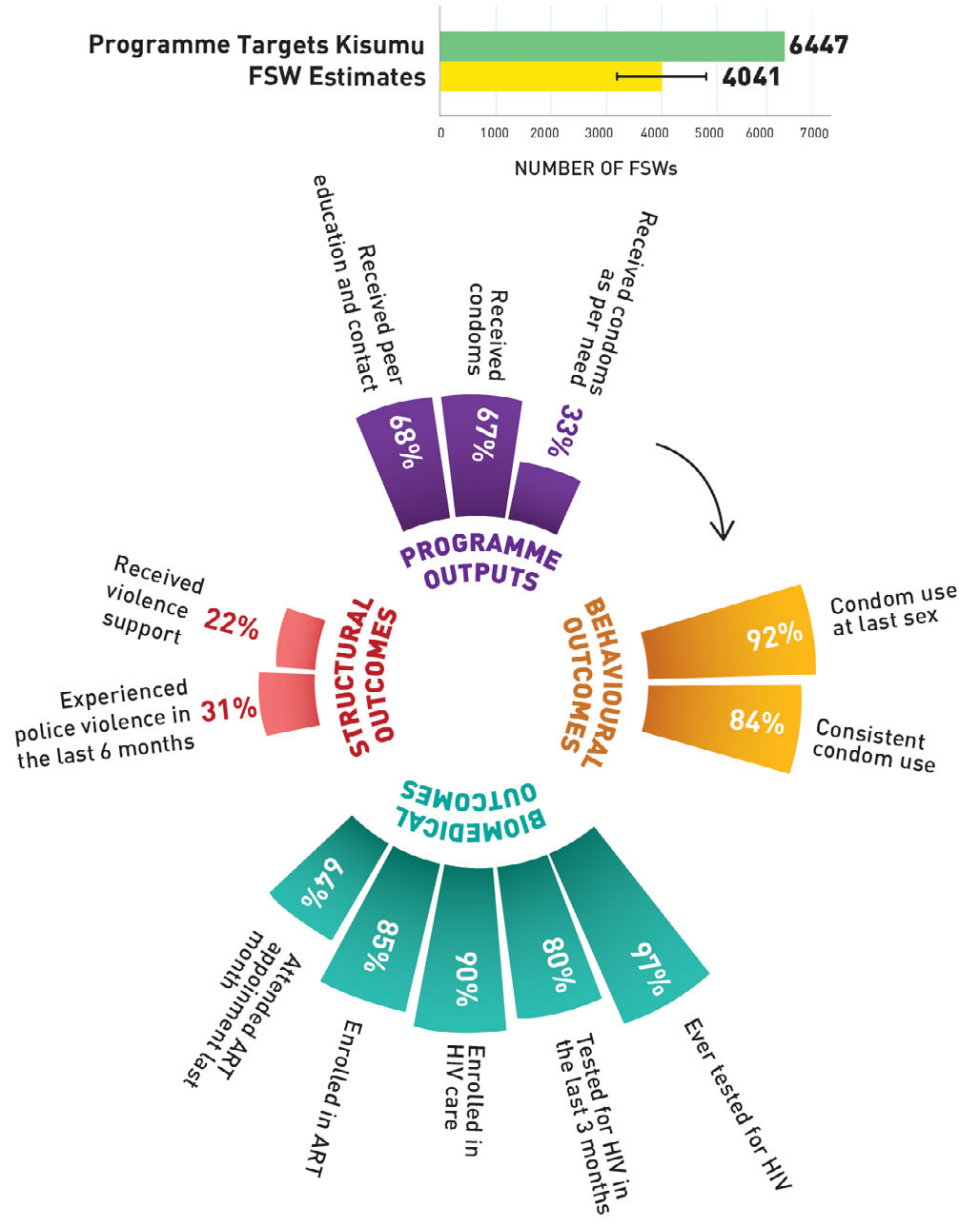
HIV Prevention programme cascade for FSW, Kisumu County, Kenya FSW, female sex worker; ART, antiretroviral treatment.

### 3.3 HIV prevention programme cascade for implementation partner at the hot spot/peer educator level

**Figure 6:**
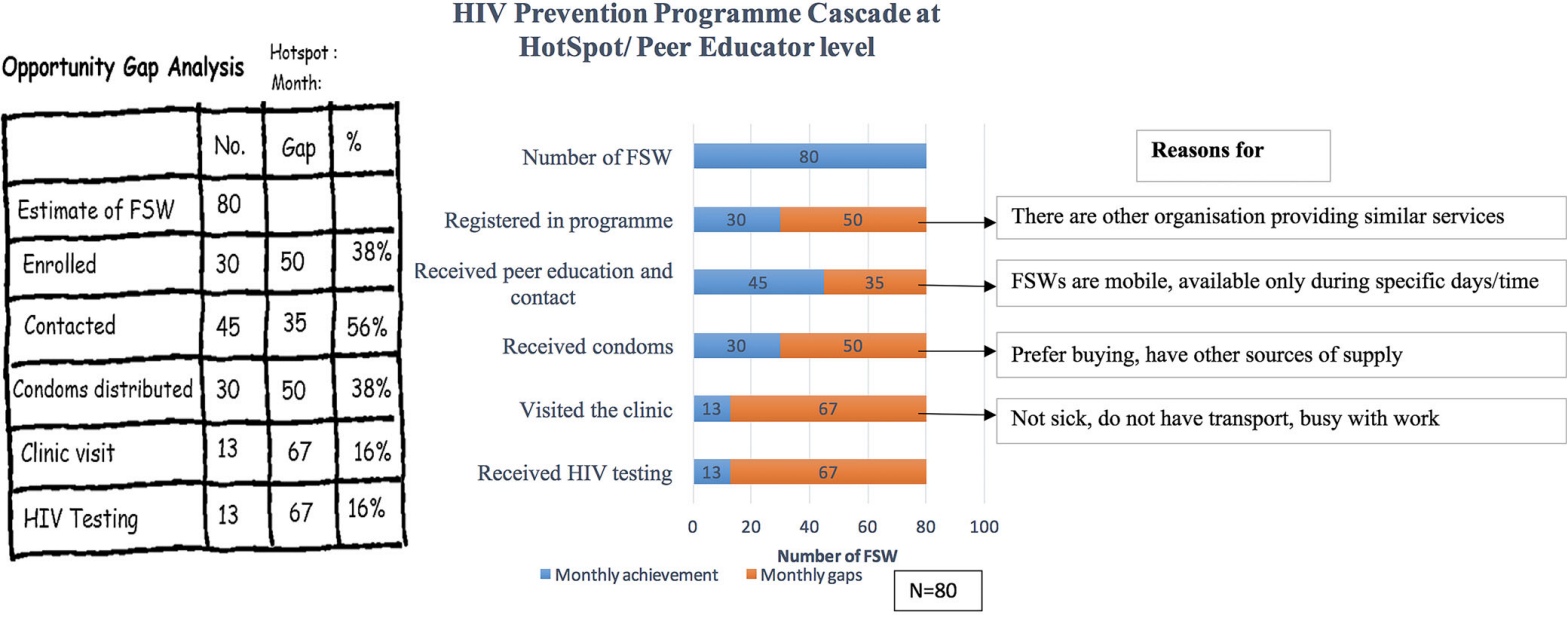
HIV prevention programme cascade for implementing partner at hot spot level, Learning site, Nairobi FSW, female sex worker.

Figure 6 shows an example of a prevention cascade at the hotspot level developed by one peer educator in Learning Site in Nairobi. In the Kenya programme, a FSW peer educator is responsible for 60 to 80 FSWs in 1 to 3 hotspots 31. As shown in Figure 6, out of the 80 FSWs who were estimated to be available in a hotspot, the peer educator was able to enrol 30 FSWs in the programme. However, in the reporting month, she met and provided information to 45 FSWs (56%) in the hotspot, 30 (38%) received condoms from her, 13 FSWs (16%) attended the clinic and all 13 received HIV testing services in the clinic. After development of this cascade, the FSW peer educator used this visual communication to assess the reasons why proportions of FSWs in the hotspot were lost at each step of programme delivery as stated in the figure.

### 3.4 HIV prevention programme cascade for implementation partner at the sub‐population level

**Figure 7:**
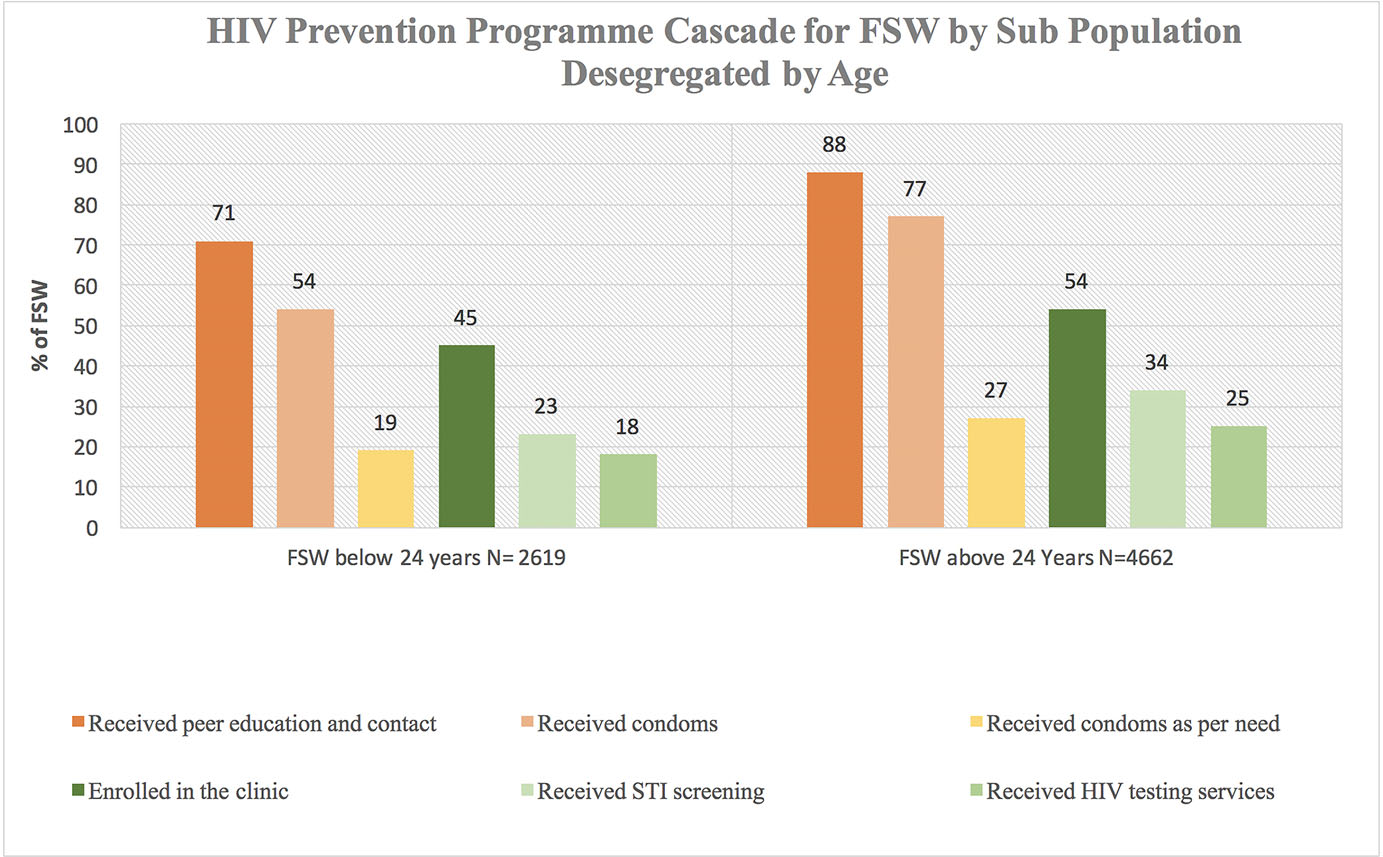
HIV prevention programme cascade for FSW, Mombasa Learning Site FSW, female sex worker; STI, Sexually Transmitted Infections.

At the implementation level, we also examined microlevel data using this approach to understand the profiles of those who were lost to follow‐up at different implementation steps. Figure 7 shows one such cascade analysis among FSWs in Learning Site in Mombasa, disaggregated by age. Out of 7281 FSWs registered in the Learning Site, 2619 (36%) were <24 years. During the reporting period, among the registered FSWs < 24 years versus those who were >24 years, we found that 71% versus 88% received peer education and contact, 54% versus 77% received condoms and 19% versus 27% received condoms as per their estimated need. 45% FSW < 24 years versus 54% of the FSWs > 24 years were enrolled in the project clinic, 23% versus 34% received STI screening and 18% versus 25% received HIV testing services.

## 4 Discussion

The recognized need for effective and efficient HIV programmes for KPs call for the development and use of effective monitoring methods that can provide timely and actionable information about programme progress and gaps 32. A combination prevention approach emphasizes a multisectoral view that includes behavioural, biomedical and structural interventions 12. Using data from Kenya, we have tried to illustrate the development and use of HIV prevention programme cascades at both the macro (national and sub‐national) and micro (implementation) levels for HIV combination prevention programmes for FSWs. The embedded analysis provides important findings for programme design, delivery and optimization.

At the national level, we found that the programme coverage targets were within the upper FSW size estimates range. Across programme outputs, there were high levels of coverage of FSWs by peer education and condom distribution (>80%) but there were also consistent gaps between the estimated need for condoms and the actual number of condoms distributed. In terms of programme outcomes, condom use at last sex act was high at 92%, (although slightly lower than the global target of 95% 33) but, levels of consistent condom use needed improvement. Evidence shows that only when need is substantially met and the supplies are adequate, then programmes can try to understand other reasons for inconsistent condom use 34. There is need for FSW programmes in Kenya to prioritize addressing condom supply and distribution gaps. Rates of HIV testing were high, but linkage to care, and ART initiation and adherence needed improvement. This emphasizes the need to devise not only differentiated ART service delivery approaches for FSWs, but also to address individual‐level and structural barriers, such as stigma, discrimination, violence and drug‐use, to initiate and adhere to ART programme 35. Experience of police violence in the past six months was high and the proportion of FSWs receiving violence support was very low, pointing to the need to strengthen structural interventions. Violence is a structural barrier that decreases the ability of FSWs to access services or adopt protective behaviours 36. An effective violence prevention and response programme not only impacts FSWs at an individual level, but improves collective agency and challenges power dynamics at the community level 37. Commonly, structural components to an HIV programme are not included in a standard HIV prevention cascades and hence not measured; yet they are critical part of a comprehensive HIV prevention programme and standard global guidance.

At the sub‐national level, the analysis of county‐level variability provides valuable information for prioritization of counties for support. While coverage of FSWs with peer education and condom distribution was high in Mombasa and Nairobi (>80%), these outputs needed improvement in Kisumu and Kiambu counties. Condoms distributed did not meet the estimated need, and this was particularly evident in Kisumu and Kiambu counties. Linkage to care and ART was poor in all counties except Kisumu. Adherence to ART was poor across the counties, and was particularly low in Mombasa. Levels of police violence were very high, with over 40% of FSWs experiencing violence in three of the counties and violence support was very low across all counties. The analysis shows that while all counties needed support in certain common areas, specific counties needed specific support like behavioural interventions in Kisumu county, linkage to ART for HIV‐positive FSWs in Mombasa and violence response mechanisms in Nairobi and Kiambu counties.

The KP Programme Manager in Kenya uses this analysis and information to guide programme improvement and design. This analysis is shared on a quarterly basis at the KP Technical Working Group and quarterly donor meetings to help the stakeholders understand programme gaps and jointly look for solutions to address the gaps. National‐level technical strategies are devised to address key gaps. Technical support is provided to the priority counties and the implementing partners from the KP Technical Support Unit (TSU) based at NASCOP as and when necessary to address the gaps.

Implementation‐level prevention programme cascades can be analysed in multiple ways. At the hotspot level, a peer educator through the cascade analysis identified the gaps to be low enrolment of the FSWs in the hotspot and lower visits to the clinic. Using the cascade, she analysed the reasons for these gaps and devised strategies to enrol more FSWs in the hotspot and motivate them to visit the clinics. Engaging frontline workers, particularly peer educators, in cascade analysis is an important step in building ownership and accountability among the frontline community staff 33, and ensuring that all KPs receive required services. Previously in India and now in Kenya, this analysis has been referred to as opportunity gap analysis 16, 18, 38.

Using a prevention programme cascade for FSW sub‐population (desegregated data of FSWs by age), the Learning Site was able to better understand the difference between sub‐populations and their access and uptake of prevention services. The analysis shows that the enrolment of FSWs below 24 years was lower in the programme generally and even among those who were enrolled, a higher proportion of them were lost at each step of service delivery. This important heterogeneity often gets missed when examining programme “averages” or overall outcomes. Our findings are similar to an analysis conducted in the “Transitions” study 39 in Mombasa, which identified that only 26% of young FSWs reported being contacted by any programme 40. This “programme access gap,” when examined further, showed that by the second year in sex work, only 15% of young FSWs had been contacted by programmes 40. The challenges of reaching young FSWs have been also highlighted in other published literature from Africa 41. This analysis highlights the importance of disaggregating data by relevant characteristics to unmask the nuanced gaps that occur in sub‐populations 42 to prioritize reaching the unreached.

HIV prevention programme cascades are useful for visualizing progress, but need to be designed to follow programme logic. Our approach measures performance and identifies gaps across HIV combination prevention programme, geographies (national, sub‐national and hotspot) and sub‐populations, by tracking inputs, key outputs and outcomes on a regular basis. This requires clear definitions of programme numerators and denominators to understand reach and coverage, and as we have demonstrated, multiple data sources can be utilized 43. Prevention programme cascades should include simple, flexible and visual tools that facilitate analysis within this framework 16. Decentralized analysis by frontline workers can lead to early identification of programme challenges and stimulate local problem‐solving 33.

A limitation to our study is that our data only allows for cross‐sectional cascades rather than cohort cascades, so we are unable to follow progression over time through the cascades. Another limitation of the study is paucity of data related to structural interventions and hence only one metric (violence) was used in this context. Lack of availability of recent KP size estimates is also a limitation as the size estimates may have been outdated in 2018. Nevertheless, there are many strengths to our approach. It uses routinely collected monitoring data from two sources to generate cascades across key outputs and outcomes for an combination HIV prevention programme with FSWs. The cascades also emphasize the need to embed an analytical process at the macro‐ and microlevels which involves managers at the national and local levels, and peer educators at the hotspot level. The tools and methods are simple to use, inexpensive, replicable, and have been applied on a large scale.

## 5 Conclusions

Prevention programme cascades serve as an effective framework to track and monitor the important programme outputs and outcomes at all levels of combination programming with FSWs. We propose in future to advance beyond linear HIV cascades to the generation and use of cascades to measure outputs and outcomes across combination prevention programming, using data from multiple sources to capture heterogeneity across prevention interventions. Adaptation of some of these learnings in the context of other KPs like MSM and PWID and another countries or programme would have to take in account the HIV epidemic, the need of the populations and context of that country or programme, so as to fit the relevant contexts and needs.

## Competing interests

The authors declare that they have no competing interests.

## Authors’ contributions

PB, HKM, MB, SM and JB conceptualized the paper. PB wrote the first draft of the paper, and HKM, MB, JB, SM, SI and SM contributed in writing different sections of the paper and reviewing the drafts. HKM and PB generated the data and managed the data collection process. JM, JK and SK supported in data collection and analysis. PB, MB, SM, SM, SKI and JB analysed various elements of the data. All co‐authors reviewed the paper and made revisions.

## Supporting information


**Table S1.** Annual programme outcome survey (polling booth survey data) HIV prevention programme cascade: national and sub‐national levels, programme outcomes (Figures 1, 2, 3, 4, 5).
**Table S2.** Routine programme monitoring data, national and sub‐national HIV prevention programme cascades: national and sub‐national levels, programme outputs (Figures 1, 2, 3, 4, 5).
**Table S3.** Routine programme monitoring data, National AIDS and STI Control Programme (NASCOP) learning site, Mombasa HIV prevention programme cascade: implementation level desegregated by age Figure 7).
