# Supplementary material for: HIV prevention programme cascades: insights from HIV programme monitoring for female sex workers in Kenya
Source: J Int AIDS Soc. 2019 Jul 22;22(Suppl Suppl 4):e25311. doi: 10.1002/jia2.25311 (PMC6643069; doi:10.1002/jia2.25311)
Supplement: Supplementary file 1 — Table S1. Annual programme outcome survey (polling booth survey data) HIV prevention programme cascade: national and sub‐national levels, programme outcomes (Figures 1, 2, 3, 4, 5). Table S2. Routine programme monitoring data, national and sub‐national HIV prevention programme cascades: national and sub‐national levels, programme outputs (Figures 1, 2, 3, 4, 5). Table S3. Routine programme monitoring data, National AIDS and STI Control Programme (NASCOP) learning site, Mombasa HIV prevention programme cascade: implementation level desegregated by age Figure 7). [file JIA2-22-e25311-s001.pdf]

Supplementary Table 1: Annual Programme Outcome Survey (Polling Booth Survey Data)

HIV Prevention Programme Cascade: National and sub national level, Programme outcomes (Figures 1, 2a,2b,2c and2d)

	**Outcome Indicators**	**Kenya = 4393**			**Mombasa = 578**			**Nairobi N=727**			**Kiambu N=319**			**Kisumu N =378**		
		**n**	**yes**	**no**	**n**	**yes**	**no**	**n**	**yes**	**no**	**n**	**yes**	**no**	**n**	**yes**	**no**
	**Behavioural Indicators**															
1	The last time you had sex with any paying client, did he use a condom?	4373	4023	350	578	531	47	725	661	64	304	278	26	378	348	30
2	During the past 1 month, was there any occasion when you had sex with any paying client without using a condom?	4347	1174	3173	572	177	395	709	153	556	313	72	241	378	62	316
	**Biomedical**															
3	Have you ever taken an HIV test?	4372	4197	175	578	565	13	710	681	29	319	308	11	378	366	12
4	Did you take an HIV test during the past 3 months?	4324	3675	649	571	485	86	719	590	129	318	284	34	367	294	73
5	If you are living with HIV, have you ever been enrolled into a HIV Care and Treatment programme (CCC) (Any service, government or private providing HIV care and treatment)?	1095	865	230	91	48	43	155	116	39	57	41	16	143	129	14
6	If you are living with HIV are you currently taking ARV (Antiretroviral drugs for HIV management)?	1095	799	296	91	43	48	155	101	54	57	37	20	143	120	23
7	Have you missed an appointment with ART clinic	1095	231	567	91	18	25	155	31	69	57	13	24	143	29	91

	and 30 days have passed since last appointment?															
	**Structural**															
8	In the past 6 months, were you ever arrested or beaten up by police and/or city askaris when you were doing sex work or at a sex work spot?	4323	2075	2248	559	242	317	717	402	315	313	170	143	377	117	260
9	In the past 6 months, when you experienced any violence, were you supported by the intervention/ clinic/ DIC? (support means medical, psychological, legal, safety/ shelter etc)	4323	1034	1033	559	96	123	717	171	237	313	83	81	377	84	61

Supplementary Table 2: Routine Programme Monitoring Data, national and sub national

HIV Prevention Programme Cascade: national and sub national level, Programme Outputs (Figures 1, 2a,2b,2c and2d)

Sl No.	Output Indicator	Kenya	Mombasa	Nairobi	Kiambu	Kisumu
	Number of Implementing Partner	92	1	3	2	2
1	Size estimates of FSW	133675	9288	29494	4603	4041
2	Programme coverage target (# of FSW targeted to reach with HIV prevention package)	174073	7328	43909	8121	6447
3	Number of FSW who received peer contact and education	156220	6134	51455	5971	4356
4	Number of FSW who received condoms	148713	6076	48077	5825	4316
5	Number of FSWs who received condoms as per need (defined as 76 condoms per FSW per month)	83053	4215	21938	2713	2130

Supplementary Table 3: Routine Programme monitoring data, NASCOP Learning Site, Mombasa

HIV Prevention programme Cascade: Implementation level desegregated by age (Figure 4)

Sl no.	Output indicators	FSW below 24 years N= 2619	FSW Above 24 Years N=4662
	Behavioural indicators		
1	Number of FSW who received peer education and contact	1878	4089
2	Number of FSW who received condoms	1438	3615
3	Number of FSW who received condoms as per need	509	1265
	Bio medical indicators		
4	Number of FSW ever enrolled in the clinic	1179	2533
5	Number of FSW who received STI screening	606	1604
6	Number of FSW who received HIV testing services	481	1178
